# Effects of virtual reality training on racket sports performance: A systematic review and meta-analysis of controlled trials

**DOI:** 10.1371/journal.pone.0345541

**Published:** 2026-04-06

**Authors:** Rui Liu, Hazwani Ahmad Yusof Hanafi, ZiMei Zhong, Rohayu Hami

**Affiliations:** 1 Department of Community Health, Cancer Research and Specialist Centre (Pusat Kanser Tun Abdullah Ahmad Badawi), Universiti Sains Malaysia (USM), Pulau Pinang, Malaysia; 2 School of Economics and Management, Hezhou University, Hezhou, Guangxi, China; Eskisehir Technical University, TÜRKIYE

## Abstract

**Background:**

Virtual reality (VR) interventions are increasingly used in racket sports, and quantitative evidence is emerging.

**Purpose:**

To synthesise controlled trials examining the effects of VR-based training on racket sports performance outcomes.

**Methods:**

A systematic search of five databases (PubMed, Web of Science, Scopus, SPORTDiscus, PsycINFO) up to 15 August 2025 was conducted following PRISMA 2020 guidelines. Eligible studies were controlled trials (randomised or non-randomised) comparing VR-based training (immersive or exergaming) with non-VR controls in tennis or table tennis players. Risk of bias was assessed using Cochrane RoB 2 (randomised) and ROBINS-I (non-randomised). Standardised mean differences (Hedges’ g) were pooled using a random-effects model with Paule–Mandel τ² and Hartung–Knapp–Sidik–Jonkman (HKSJ) adjustment. The protocol was registered (PROSPERO CRD420251132325).

**Results:**

Six controlled trials(total N = 426; analysed N = 401) were included. The pooled meta-analysis indicated a moderate overall effect favouring VR (Hedges’ g = 0.78; HKSJ 95% CI [0.41, 1.15]), with moderate heterogeneity (I² = 52%). The 95% prediction interval was [−0.01, 1.57], which spans the null effect, indicating that in some future populations or settings VR training may not yield a meaningful performance advantage. One trial investigated perceptual-cognitive VR training (Anguera et al., 2025), showing a substantial but imprecise effect (g = 0.81, 95% CI [−0.05, 1.67]). As only a single study was available, this evidence is presented descriptively rather than meta-analytically. A subgroup examination of five physically engaging VR interventions found a similarly large effect (*g* = 0.78, 95% CI [0.31, 1.25]) despite considerable heterogeneity (I² = 62%).

**Conclusion:**

According to six controlled trials, VR training was associated with average performance enhancements in racket sports; however, the 95% prediction interval [−0.01, 1.57] encompasses the null effect, indicating that a future study could plausibly show no benefit. Due to the limited evidence base (k = 6), significant heterogeneity (I² = 52%), and considerable statistical uncertainty, the existing evidence does not allow for definitive conclusions regarding the efficacy of VR. The current findings should be regarded as a preliminary signal rather than confirmation of effectiveness. Larger, more methodologically robust RCTs with standardised outcomes are needed before definitive recommendations can be made.

## 1. Introduction

Virtual reality (VR) has transitioned from a niche technology to a mainstream performance tool in sport. Recent reviews report growing adoption of VR across various sports domains–including invasion, striking, and precision sports–for skill acquisition, assessment, and rehabilitation, with generally positive but mixed effects on learning and performance [1–4]. These syntheses consistently highlight critical limitations within the literature: small sample sizes, heterogeneous tasks and outcome measures, and insufficient testing of skill transfer to real-world performance–underscoring the need for sport-specific meta-analyses [1–4]. For the purposes of this review, the term “VR” is used as an umbrella term encompassing immersive VR (e.g., head-mounted displays) and physically active video games (exergames) designed for racket sports training.

A strong theoretical rationale underpinning VR’s potential in sport stems from established motor learning principles [5,6] and contemporary frameworks like ecological dynamics and Representative Learning Design (RLD) [7,8]. These perspectives argue that effective training must preserve the critical information-movement couplings inherent in competition, ensuring perception and action remain functionally linked. VR offers unprecedented control over key contextual constraints (e.g., opponent kinematics, ball flight dynamics, tactical scenarios), enabling high-volume, repeatable exposure to the specifying cues essential for anticipation and decision-making under pressure [9,10]. Foundational RLD work and contemporary practice-design scholarship further suggest that representativeness exists on a continuum that practitioners can strategically modulate across micro- and meso-cycles, moving beyond a simplistic “VR vs. real” dichotomy [8–10]. Crucially, racket sport-specific representativeness studies demonstrate that VR environments can effectively approximate competition-relevant informational variables, supporting the core RLD premise within these sports [11].

Regarding perceptual-cognitive training specifically (e.g., reading serves, anticipating shot direction, recognizing tactical patterns), VR scoping reviews indicate promising effects, particularly when tasks faithfully reproduce competitive informational constraints and demand time-pressured decisions—situations where achieving sufficient repetition on-court can be logistically challenging or prohibitively expensive [4,12]. These reviews also emphasize the need for improved reporting of task representativeness, clearer outcome taxonomies, and more robust tests of transfer to actual performance. Simultaneously, translational reviews caution that fidelity in VR is multifaceted, encompassing perceptual, task/functional, and action/haptic dimensions [3,13]. Benefits are most pronounced when perceptual and task fidelity are high. Conversely, limitations in action/haptic fidelity (e.g., the absence of authentic racket-ball contact forces and proprioceptive feedback) may constrain the transfer of gains for purely motor outputs or general physical abilities [3,13]. This helps explain why VR may be particularly impactful for training perception and decision-making, while its effects on fundamental motor skills like balance might be more modest without addressing haptic realism.

These converging theoretical and empirical lines motivate a racket sports-specific quantitative synthesis. Racket sports like tennis and table tennis are quintessential open-skill sports characterized by severe temporal pressure, demanding rapid integration of anticipation, decision-making, and whole-body movement execution. Existing VR trials in racket sports vary considerably in their focus (cognitive tasks vs. physically active applications), intervention dosage, and outcome measures (ranging from competitive ratings like Universal Tennis Rating (UTR) to reaction times and balance scores), leaving practitioners uncertain about contexts where VR provides the most significant value. This systematic review and meta-analysis therefore aims to: (1) quantify the overall effect of VR training on racket sports performance outcomes compared with non-VR controls, and (2) to examine whether effects differ between perceptual-cognitive and physically active VR interventions.

## 2. Methods

### 2.1. Protocol and registration

This systematic review and meta-analysis was conducted and reported in accordance with the Preferred Reporting Items for Systematic Reviews and Meta-Analyses (PRISMA) 2020 statement [14]. The study protocol was prospectively registered with the International Prospective Register of Systematic Reviews (PROSPERO) under registration number CRD420251132325，and publicly accessible at: https://www.crd.york.ac.uk/prospero/display_record.php?ID=CRD420251132325.

### 2.2. Eligibility criteria

Studies were selected based on the pre-specified PICOS framework:

**Population (P):** Adults and children who engage in competitive or recreational racket sports (tennis or table tennis), regardless of age, sex, and the level of expertise.

**Intervention (I):** Any type of training programme employing mainly immersive VR (e.g., head-mounted displays - HMDs) or physically active video games (exergames) specifically designed to enhance racket sports.

**Comparator (C):** A control condition without VR. This may be a passive control (no further training, waiting list) or an active control (e.g., traditional on-court practice, non-VR forms of training).

**Outcomes (O):** Quantitative measures of racket sports performance or validated proxies (e.g., competition metrics such as UTR; sport-specific anticipation/decision tasks; reaction time; dynamic balance where justified). Studies reporting only subjective measures (e.g., enjoyment, presence) or only physiological outcomes without a clear performance link were excluded.

**Study design (S):** Controlled trials (randomised and nonrandomised trials), parallel-group and crossover designs. Non-controlled studies, case reports, reviews, and conference abstracts without detailed methods or results were ineligible.

### 2.3. Search strategy

A comprehensive literature search was performed in the following five electronic databases from their inception to August 15, 2025: PubMed, Web of Science (Core Collection), Scopus, SPORTDiscus (via EBSCOhost), and PsycINFO (via Ovid). The search strategy employed a combination of keywords and controlled vocabulary terms (e.g., MeSH in PubMed, Thesaurus in PsycINFO) related to three core concepts: 1) Sport: (tennis OR table tennis OR “racket sports”); 2) Intervention: (“virtual reality” OR VR OR “head-mounted display” OR HMD OR exergame OR “active video game”); 3) Study Design: (“controlled trial” OR RCT OR “randomised” OR “randomly assigned” OR “clinical trial”). Boolean operators (AND, OR) and appropriate truncation symbols were used. The search strategy was peer-reviewed using the PRESS checklist [13] and adapted for each database. No language restrictions were applied. Additionally, reference lists of all included studies and relevant systematic reviews were manually screened to identify potentially eligible articles missed by the electronic search.The complete Boolean search strings for all databases are provided in S1 Table.

### 2.4. Study selection and data extraction

Search results were imported into reference management software (e.g., EndNote) for de-duplication. Automatic and manual deduplication was performed; no duplicates were detected across the five databases for the specific search terms used. Two reviewers independently screened titles and abstracts against the eligibility criteria. Full texts of potentially relevant records were then assessed independently by both reviewers; disagreements were resolved by discussion or consultation with a third reviewer. Data were extracted using a piloted form capturing study characteristics, participants, intervention/comparator details, outcomes and assessment timing, results (means, standard deviations (SDs), change scores, and p-values where available). One reviewer performed extraction and a second verified accuracy. For multi-arm studies, conceptually similar VR arms were combined to create a single VR–control comparison to avoid unit-of-analysis error, following Cochrane guidance.

### 2.5. Risk of bias assessment

Two reviewers independently assessed randomised trials using RoB 2 [15] across five domains (randomisation process; deviations from intended interventions; missing outcome data; outcome measurement; selection of the reported result), with judgements of low risk, some concerns, or high risk. Non-randomised studies were assessed using ROBINS-I with low, moderate, serious, or critical risk of bias. Discrepancies were resolved by discussion. Tool-specific, domain-level results are provided in S1, S2 and S4 Figs; here we report study-level overall judgements.

### 2.6. Statistical analysis

Hedges’ g was used to adjust for small-sample bias in calculating the standardized mean difference (SMD), since this was the primary measure of effect [16]. In any given study, the effect size for the primary performance outcome was calculated by comparing the average post-intervention scores of the VR group and control group. When studies reported more than one performance outcome, we used a pre-defined outcome hierarchy: (1) sport-specific skill composites or competitive ratings (e.g., skill batteries, UTR) were given more weight than isolated motor proxies; (2) outcomes measured in game-like situations were given more weight than laboratory measures; and (3) when there were still multiple outcomes at the same hierarchy level, the outcome that was most commonly reported across studies was chosen to make comparison easier. This hierarchy was created to make sure that the outcomes we chose were in line with the idea of “racket sports performance” that we used to decide who was eligible. For studies with multiple intervention arms compared to one control group, the most relevant VR intervention arm (giving preference to immersive VR and focusing on the main components of sport skill) was included in the primary analysis to ensure that the control group would not be double-counted. Conceptually similar VR arms were combined into a single VR group-to avoid double-counting--when there were multiple VR arms and only one control. We pre-specified one main VR priority arm (immersive and sport-skill focused) for the analysis, in accordance with Cochrane guidance. For smaller-is-better metrics (e.g., reaction time), we reversed the sign to make positive SMD favour VR. For effect sizes without standard metrics, we transformed them into Cohen’s d and corrected to Hedges’ g where necessary; this conversion was applied after justification and is described in S1 Table. Standard errors for Hedges’ g were calculated as in the original paper [17]. Effect sizes were combined using a random-effects meta-analysis model which accounts for both within-study and between-study heterogeneity, using the Paule–Mandel estimator for τ² (i.e., between-study variance). We used the Hartung-Knapp-Sidik-Jonkman (HKSJ) method to adjust the p-values and confidence intervals (CIs) for the pooled estimate. HKSJ uses a t-distribution with k − 2 degrees of freedom instead of a normal distribution, which gives wider and more appropriate confidence intervals when the number of studies is small, as in this review [18,19]. This adjustment provides substantially better control of Type I error rates when k < 10 [18]. We calculated prediction intervals using the identical t_{k − 2} quantile, recognising that with k = 6, these intervals are inherently imprecise.Small-study tests (e.g., Egger’s) were not performed because k = 6. Analyses were conducted in R (R Foundation) using the meta package with Paule–Mandel τ² (method.tau = “PM”) and HKSJ adjustment (hakn = TRUE). The I² statistic was used to quantify heterogeneity among study effects, with the following interpretation: 0–40% might not be important; 30–60% may represent moderate heterogeneity; 50–90% may represent sizable heterogeneity; 75–100% equals considerable heterogeneity [20]. We also estimated the prediction interval, which indicates the range within which the true effect size of a future study (necessarily random) would be expected to fall [20,17]. An exploratory subgroup analysis was always planned to explore the possibility of different effect sizes based on which aspect of VR intervention was primary focus: Perceptual-Cognitive VR (interventions primarily aimed at anticipation, decision-making, pattern recognition, or tactical understanding using cognitive tasks within VR) and Physically Active VR (interventions mainly focused on motor skills, coordination, balance, or reaction time using exergames or physically engaging VR scenarios that require significant body movement). In a secondary exploratory sensitivity analysis, we compared sport-specific skill outcomes (e.g., skill batteries, UTR ranking) with general motor proxies (e.g., reaction time, dynamic balance). We performed sensitivity analyses to check the robustness of our results, which included conducting a leave-one-out analysis for each study (with the pooled estimate recalculated). We used visual inspection of a funnel plot to perceive publication bias, acknowledging its limited power due to the small number of studies (k = 6). Because k = 6, small-study tests (e.g., Egger’s) were not performed, and the funnel plot was interpreted qualitatively only.

### 2.7. Ethical considerations

No ethical approval was required for this study as it synthesised data from previously published studies. The study protocol was prospectively registered with PROSPERO (CRD420251132325).

## 3. Results

### 3.1. Study selection

**Fig 1:**
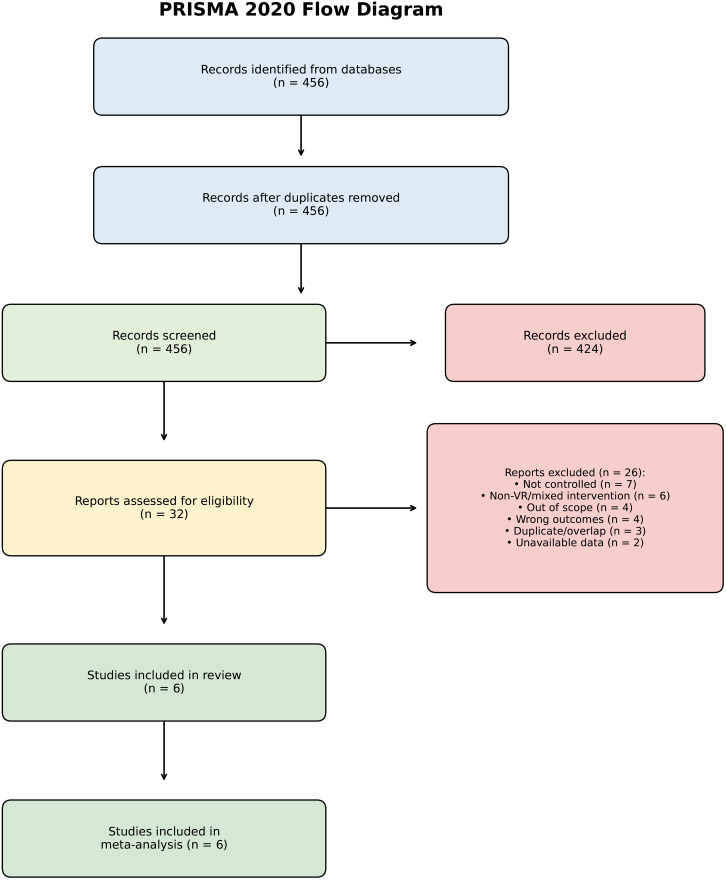
PRISMA 2020 flow diagram illustrating the study selection process. Records were identified from five electronic databases (PubMed, Web of Science, Scopus, SPORTDiscus, PsycINFO; total n = 456). After title/abstract screening and full-text assessment, six controlled trials met eligibility criteria and were included in the systematic review and meta-analysis.

After deduplication, the systematic search retrieved 456 records in total. After title and abstract screening, 32 full-text articles were reviewed for eligibility. The inclusion criteria were met by six controlled trials and they were selected for meta-analysis. A flow diagram depicting the study selection process according to PRISMA is presented in Fig 1.

### 3.2. Study characteristics

Six controlled trials were included (overall N = 426; primary meta-analysis N = 401: VR n = 200; control n = 201). Three studies focused on tennis [21–23] and three on table tennis [24–26]. One study used a perceptual-cognitive intervention [23], while five used physically active interventions [21,22,24–26]. Four studies employed short-term interventions (single or a few sessions) [21,22,24,26]; two implemented 10–12-week programmes [23,25]. Controls varied (passive or active). Participants ranged from adolescents to adults with novice-to-intermediate skill levels. Outcomes included dynamic balance, reaction time, composite skill scores, and competitive ratings (e.g., UTR). Table 1 summarises study characteristics (sport, design, N, intervention type, outcomes, effect sizes, and overall RoB).

**Table 1:** Characteristics of included studies. RCT = randomised controlled trial; Non-RCT = non-randomised controlled trial; VR = virtual reality; UTR = Universal Tennis Rating; RoB = overall risk of bias (RoB 2 for RCTs; ROBINS-I for non-RCTs). Effect sizes are Hedges’ g with HKSJ-adjusted 95% confidence intervals.

Study	Design	N	VR Type	Primary Outcome	Hedges’ g [95% CI]	RoB
Michalski et al. (2019)	RCT	57	Physically Active	Skill composite	g = 1.08 [0.53, 1.63]	Some concerns
Ma et al. (2024)	RCT	120	Physically Active	Skill composite	g = 1.10 [0.71, 1.49]	Low
Novak et al. (2023)	Non-RCT	58	Physically Active	Dynamic balance	g = 0.54 [0.15, 0.93]	High
Flôres et al. (2024)	RCT	105	Physically Active	Reaction time	g = 0.23 [−0.26, 0.72]	Some concerns
Škopek et al. (2024)	Non-RCT	38	Physically Active	Skill battery	g = 0.96 [0.47, 1.45]	Some concerns
Anguera et al. (2025)	RCT	23	Perceptual-Cognitive	UTR rating	g = 0.81 [−0.05, 1.67]	Low

### 3.3. Risk of bias

Two studies had a low overall risk of bias, three raised some concerns, and one had a high risk. This was primarily due to factors inherent in physical training studies (e.g., lack of blinding, deviations from intended interventions, and missing outcome data). RoB 2 judgments are presented as Low/ Some concerns/ High. ROBINS-I judgments are presented as Low/ Moderate/ Serious/ Critical. Domain-specific, tool-specific results are provided in S1 and S2 Figs included with this article.

### 3.4. Overall meta-analysis

**Fig 2:**
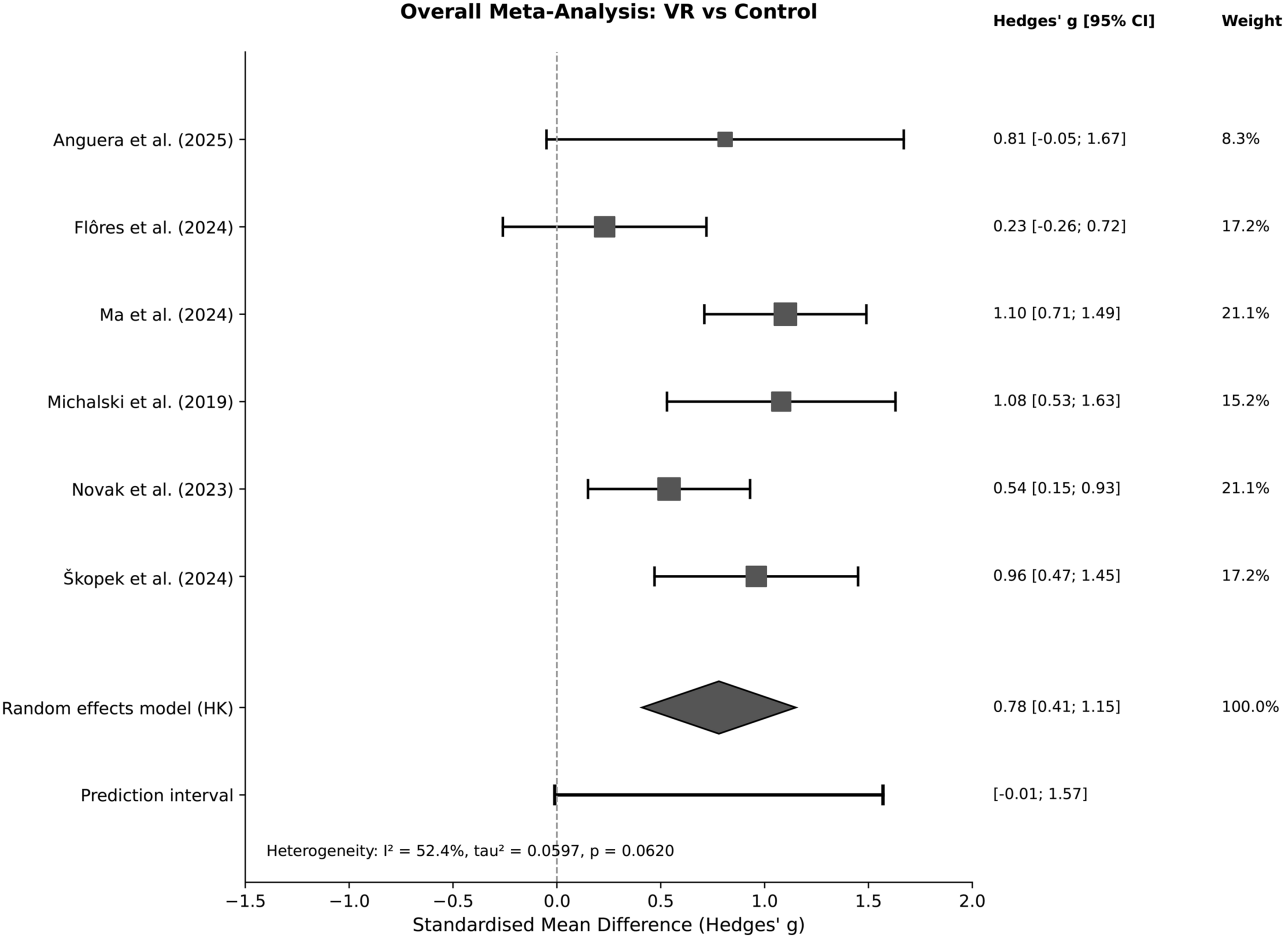
Forest plot of the overall meta-analysis comparing VR-based training with non-VR controls on racket sports performance outcomes (k = 6 studies; N = 401). Effect sizes are Hedges’ g with Hartung–Knapp–Sidik–Jonkman (HKSJ) adjusted 95% confidence intervals. The pooled estimate was g = 0.78 (95% CI [0.41, 1.15]). The 95% prediction interval [−0.01, 1.57] is shown as a horizontal bar at the bottom of the plot. Heterogeneity: I² = 52.4%, τ² = 0.0597, p = 0.062. Square size is proportional to study weight; horizontal lines represent 95% CIs; the diamond represents the pooled estimate.

Using the Paule–Mandel τ² estimator with HKSJ adjustment, the pooled SMD was g = 0.78 (95% CI [0.41, 1.15]), with I² = 52% (τ² = 0.060). The 95% prediction interval was [−0.01, 1.57] (Fig 2). Critically, this interval spans the null, indicating that in some future populations or contexts VR training may not yield a meaningful performance advantage—a result that highlights the considerable uncertainty surrounding the overall estimate and argues against overconfident interpretation.

### 3.5. Subgroup analysis

**Fig 3:**
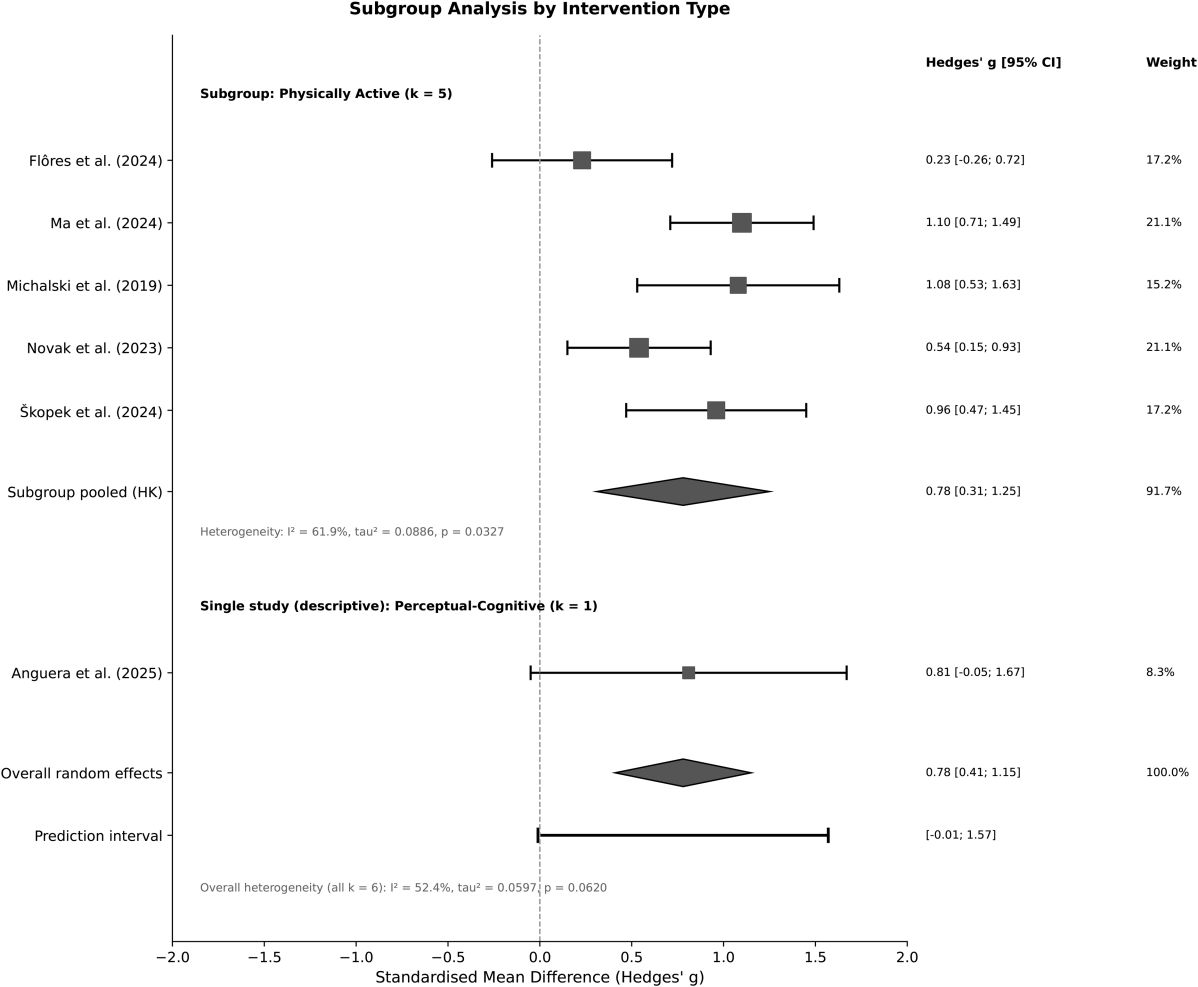
Forest plot of the subgroup analysis by intervention type. The Physically Active VR subgroup (k = 5) yielded a pooled effect of g = 0.78 (95% CI [0.31, 1.25]; I² = 61.9%). The single Perceptual-Cognitive VR study (Anguera et al., 2025; k = 1) is presented descriptively only (g = 0.81, 95% CI [−0.05, 1.67]); no heterogeneity statistics are reported for this single-study subgroup. Overall heterogeneity statistics (all k = 6) are reported at the bottom of the figure. Effect sizes are Hedges’ g with HKSJ-adjusted 95% confidence intervals.

Fig 3 shows the planned subgroup analysis by type of intervention. Perceptual-cognitive VR underwent evaluation in a singular trial (Anguera et al., 2025); a meta-analysis is infeasible with k = 1, thus we present its effect descriptively: g = 0.81 (95% CI [−0.05, 1.67]). The confidence interval is broad and includes zero, preventing definitive conclusions regarding this type of intervention. For the five physically active VR interventions, the combined effect was g = 0.78 (95% CI [0.31, 1.25]), with a significant level of variation (I² = 61.9%, τ² = 0.089). These subgroup findings should be regarded as exploratory due to the insufficient number of studies, and no formal test for subgroup differences was meaningful given the single-study subgroup.

### 3.6. Sensitivity analysis and risk of bias assessment

**Fig 4:**
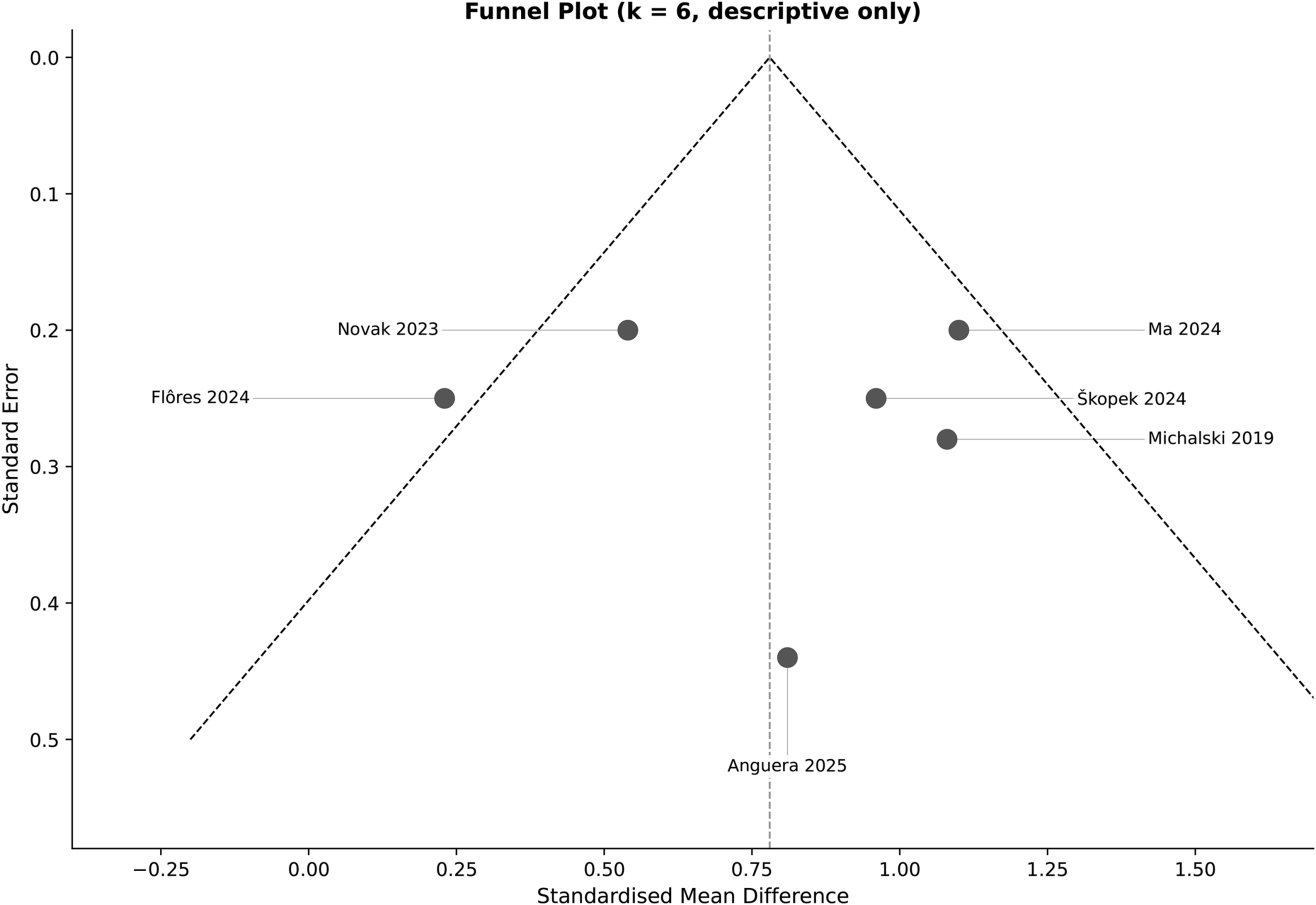
Funnel plot of standardised mean difference (Hedges’ g) against standard error for all included studies (k = 6). The plot is presented for descriptive purposes only; with k = 6 studies, formal tests for small-study effects (e.g., Egger’s test) lack sufficient power and were not performed. Visual inspection suggests slight asymmetry, but no definitive conclusions regarding publication bias can be drawn from this plot alone.

A sensitivity analysis restricted to sport-specific skill outcomes—Anguera, Michalski, Ma, and Škopek [22–26]—yielded a similarly positive but more precise pooled estimate (g = 0.92, 95% CI [0.41, 1.43]). In contrast, studies assessing general motor proxies—Novak and Flôres [21,22]—showed a small, non-significant effect (g = 0.23, 95% CI [−0.26, 0.72]). Leave-one-out analyses (S3 Fig) did not reverse the positive direction of the overall effect. The funnel plot (k = 6) showed some asymmetry, but publication bias cannot be meaningfully assessed with so few studies (Fig 4).

## 4. Discussion

### 4.1. Principal findings in context

Pooling results from six controlled trials involving 426 participants (primary meta-analysis N = 401) indicated a moderate-to-large overall effect favouring VR, albeit with considerable heterogeneity. Nevertheless, considerable heterogeneity and wide uncertainty bands (HKSJ CIs and the prediction interval) caution against overgeneralisation. Subgroup patterns were consistent with theory: the sole perceptual--cognitive trial suggested a potentially large effect but was imprecise, whereas physically active interventions showed a moderate‐to‐large average effect amid high heterogeneity. This pattern aligns strongly with broader reviews of VR in sport, which consistently report the most robust evidence for applications where technology preserves the critical informational constraints of competition and demands time-pressured decisions–core tenets of Representative Learning Design (RLD)–rather than attempting to substitute high-fidelity motor interactions like racket-ball contact [1,3,4,9].

The analysis of these results must also consider the methodological rigour of the studies involved. Only two trials (Ma et al. and Anguera et al.) showed a low overall risk of bias. Three studies raised some concerns, and one (Novak et al.) was considered high risk because it lacked blinding and there was a chance that the interventions would not be carried out as planned. Because it is hard to blind participants and staff in studies of physical training, performance bias may inflate observed effect sizes. Additionally, non-randomized designs (three out of six studies) may amplify treatment effects due to unmeasured confounding variables. These methodological limitations underscore the necessity for prudent interpretation of the aggregated estimate.

### 4.2. Intervention type and potential mechanisms

The sole perceptual-cognitive study reported a large but imprecise effect, consistent with the premise that VR can target decision-making under representative informational constraints; nonetheless, inference is limited by k = 1. Physically active VR produced a large average effect but was accompanied by high heterogeneity (I² = 62%).

### 4.3. Sources of heterogeneity and practical implications

The moderate-to-substantial heterogeneity observed in this review (I² = 52% overall; 62% in the physically active subgroup) likely reflects multiple sources of variability that warrant careful consideration.

Initially, outcome type surfaced as a principal factor influencing heterogeneity. The sensitivity analysis demonstrated a significant disparity: sport-specific skill outcomes (g = 0.92, 95% CI [0.41, 1.43]) exhibited considerably greater effects compared to general motor proxies, including dynamic balance and reaction time (g = 0.23, 95% CI [−0.26, 0.72]). This pattern has significant ramifications for both theoretical frameworks and practical applications. From the standpoint of Representative Learning Design (RLD), this finding aligns with the principle that transfer is optimised when training tasks and assessment results exhibit essential informational constraints analogous to the target performance environment.

Second, and most importantly, our broad definition of “VR” conflated interventions that had very different fidelity levels. Immersive VR systems (e.g., head-mounted displays with motion tracking) and commercial exergames exhibit significant differences in their maintenance of perception-action coupling, which is the fundamental principle of ecological dynamics and RLD theory. Most commercial exergames lack haptic fidelity and may distort kinematic patterns to register game inputs, potentially violating the information-movement couplings that RLD posits as essential for representative practice. Our analysis may have obscured differential effects that the theoretical framework we used would have predicted by combining these different types of interventions. This represents a key limitation because the current evidence base does not allow us to rigorously test whether high-fidelity immersive VR systems are better than lower-fidelity exergaming approaches, as RLD would suggest.

For practitioners, the available evidence tentatively suggests VR may be most useful when targeting sport-specific skill outcomes rather than general motor abilities; however, this should be treated as a working hypothesis rather than an established recommendation. VR should be considered a complement to—rather than replacement for—on-court/table practice, particularly for high-repetition training of perceptual-cognitive demands where achieving sufficient volume in live settings is logistically challenging. Coaches and sport scientists should carefully evaluate the fidelity characteristics of available VR systems, prioritising those that preserve critical perception-action couplings relevant to their sport context. Given that the 95% prediction interval includes zero, the limited evidence base (k = 6), and the methodological weaknesses of primary studies, practitioners should treat current findings as a preliminary signal only, remain cautious about substantial investments in VR infrastructure, and await confirmation from larger, higher-quality RCTs before incorporating VR training as a standard practice component.

### 4.4. Limitations and future research

Major limitations include the small evidence base (k = 6), predominance of short-term interventions (4 of 6 studies used single or few sessions), lack of long-term follow-up, and limited demographic diversity (predominantly male, novice-to-intermediate participants). The absence of elite athlete samples limits generalizability to high-performance contexts. Substantial heterogeneity and insufficient power precluded meaningful publication bias assessment. We did not perform a formal certainty-of-evidence (GRADE) assessment due to the exceedingly limited evidence base (k = 6) and significant clinical and methodological heterogeneity, which would have precluded meaningful GRADE ratings beyond ‘very low certainty’ for all outcomes. This limitation is noted in the PRISMA 2020 checklist (Item 15).

## 5. Conclusions

This meta-analysis of six controlled trials revealed that VR-based training was associated with average enhancements in racket sports performance (g = 0.78). However, this conclusion must be interpreted with substantial caution: as shown in Section 3.4, the prediction interval spans the null, leaving open the possibility that VR training may yield no benefit in some future contexts. The pooled estimate reflects low overall certainty owing to a minimal evidence base (k = 6), moderate-to-substantial heterogeneity (I² = 52%), significant imprecision, and methodological weaknesses in primary studies. Consequently, the current findings represent a preliminary signal that warrants further investigation rather than confirmation of effectiveness. Definitive conclusions about the efficacy of VR training for racket sports cannot be drawn from the existing evidence; high-quality, adequately powered RCTs with standardised sport-specific outcomes are needed before evidence-based recommendations can be made.

Evidence is most consistent for sport-specific skill outcomes. VR should be considered a complement to on-court/table practice—particularly for high-repetition training of perceptual-cognitive demands. Larger, longer-term, methodologically robust RCTs with standardised, sport-specific outcomes are needed to define efficacy and best-practice implementation.

## Supporting information

S1 Fig Risk of bias domain-level heatmap (RoB 2). Domain-level risk of bias assessments for each randomised trial across the five RoB 2 domains: randomisation process, deviations from intended interventions, missing outcome data, outcome measurement, and selection of the reported result. Judgements: Low (green), Some concerns (yellow), High (red).
(PDF)

S2 Fig Risk of bias domain-level heatmap (ROBINS-I). Domain-level risk of bias assessments for each non-randomised controlled trial using the ROBINS-I tool across seven domains. Judgements: Low (green), Moderate (yellow), Serious (orange), Critical (red).
(PDF)

S3 Fig Leave-one-out sensitivity analysis. Forest plot showing the pooled effect estimate (Hedges’ g) and 95% confidence and prediction intervals when each study is omitted in turn. The overall direction of effect remained positive and statistically significant across all iterations, indicating robustness of the pooled estimate.
(PDF)

S4 Fig Risk of bias summary — overall judgement per study. Bar chart displaying the overall risk of bias judgement for each of the six included studies: Low risk (green), Some concerns (orange), High risk (red). Assessed using RoB 2 for randomised trials and ROBINS-I for non-randomised controlled trials.
(PDF)

S1 Table Complete Boolean search strategies. Full electronic search strings for all five databases (PubMed, Web of Science, Scopus, SPORTDiscus, PsycINFO) with last-searched dates (15 August 2025) and number of records retrieved per database.
(PDF)

S1 File PRISMA 2020 checklist. Completed PRISMA 2020 reporting checklist indicating the location of each required reporting item within the manuscript. Items 15 and 22 (GRADE certainty assessment) are marked as not performed; rationale is provided in Section 4.4.
(PDF)

## Abbreviations

CI: Confidence Interval
CRediT: Contributor Roles Taxonomy
HKSJ: Hartung–Knapp–Sidik–Jonkman
HMD: Head-Mounted Display
I²: I-squared statistic
N: Number of participants
O: Outcomes
P: Population
PICOS: Population, Intervention, Comparator, Outcomes, Study design
PM: Paule–Mandel estimator
PRISMA: Preferred Reporting Items for Systematic Reviews and Meta-Analyses
PROSPERO: International Prospective Register of Systematic Reviews
RCT: Randomised Controlled Trial
RLD: Representative Learning Design
RoB 2: Risk of Bias 2
ROBINS-I: Risk of Bias in Non-randomised Studies – of Interventions
S: Study design
SD: Standard Deviation
SMD: Standardised Mean Difference
UTR: Universal Tennis Rating
VR: Virtual Reality
