# Supplementary material for: Effects of virtual reality training on racket sports performance: A systematic review and meta-analysis of controlled trials
Source: PLoS One. 2026 Apr 6;21(4):e0345541. doi: 10.1371/journal.pone.0345541 (PMC13052862; doi:10.1371/journal.pone.0345541)
Supplement: S1 Fig — Domain-level risk of bias assessments for each randomised trial across the five RoB 2 domains: randomisation process, deviations from intended interventions, missing outcome data, outcome measurement, and selection of the reported result. Judgements: Low (green), Some concerns (yellow), High (red). (PDF) [file pone.0345541.s001.pdf]

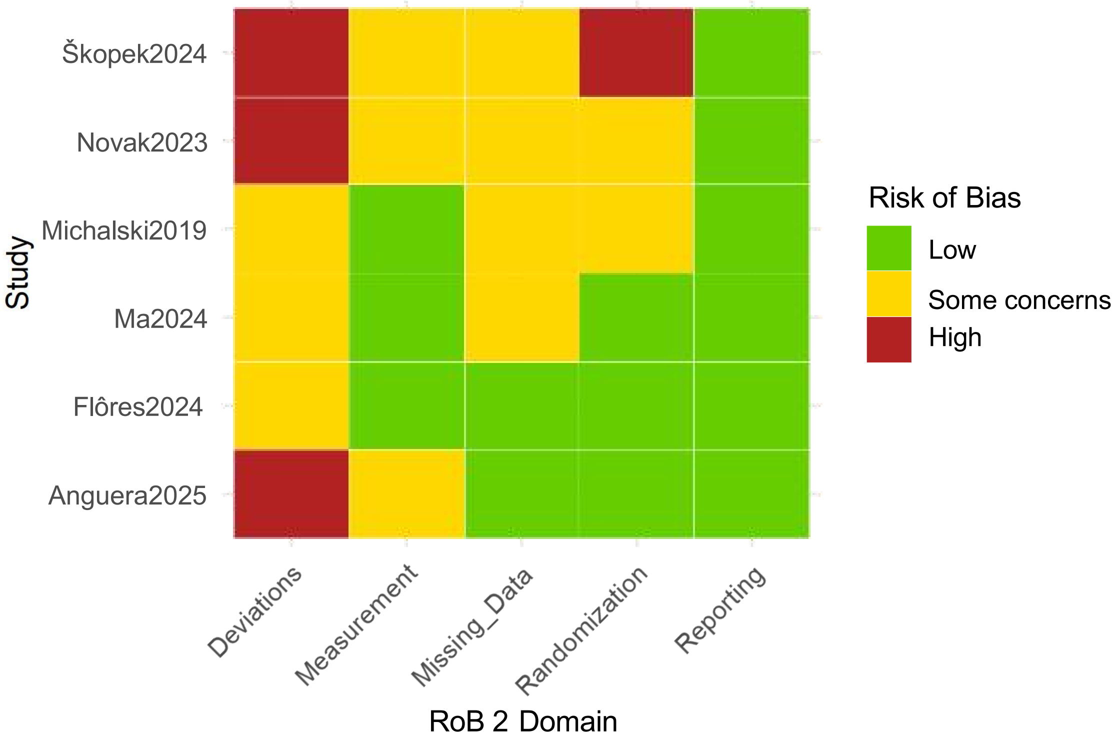
Škopek2024
Novak2023
Michalski2019
Ma2024
Flôres2024
Anguera2025
Study
Deviations
Measurement
Missing_Data
Randomization
Reporting
RoB 2 Domain
Risk of Bias
Low
Some concerns
High
