# Supplementary material for: Effects of virtual reality training on racket sports performance: A systematic review and meta-analysis of controlled trials
Source: PLoS One. 2026 Apr 6;21(4):e0345541. doi: 10.1371/journal.pone.0345541 (PMC13052862; doi:10.1371/journal.pone.0345541)
Supplement: S2 Fig — Domain-level risk of bias assessments for each non-randomised controlled trial using the ROBINS-I tool across seven domains. Judgements: Low (green), Moderate (yellow), Serious (orange), Critical (red). (PDF) [file pone.0345541.s002.pdf]

S2 Fig. Risk of Bias Domain-Level Assessment (ROBINS-I)

Non-randomised controlled trials: Novak et al. (2023) and Škopek et al. (2024)

Study	Confounding	Selection of Participants	Classification of Interventions	Deviations from Intended Interventions	Missing Data	Measurement of Outcomes	Selection of Reported Result
**Novak et al. (2023)**	Moderate	Low	Low	Serious	Low	Moderate	Low
**Škopek et al. (2024)**	Moderate	Low	Low	Moderate	Low	Moderate	Low

Legend: Low (green) Moderate (yellow) Serious (orange) Critical (red)

Note: Risk of bias assessed using ROBINS-I (Risk Of Bias In Non-randomised Studies – of Interventions). Domains assessed: (1) Confounding, (2) Selection of participants, (3) Classification of interventions, (4) Deviations from intended interventions, (5) Missing data, (6) Measurement of outcomes, (7) Selection of the reported result. Judgements: Low, Moderate, Serious, Critical.
