# Supplementary figures and images for: Effects of virtual reality training on racket sports performance: A systematic review and meta-analysis of controlled trials
Source: PLoS One. 2026 Apr 6;21(4):e0345541. doi: 10.1371/journal.pone.0345541 (PMC13052862; doi:10.1371/journal.pone.0345541)

# Leave-One-Out Meta-Analysis

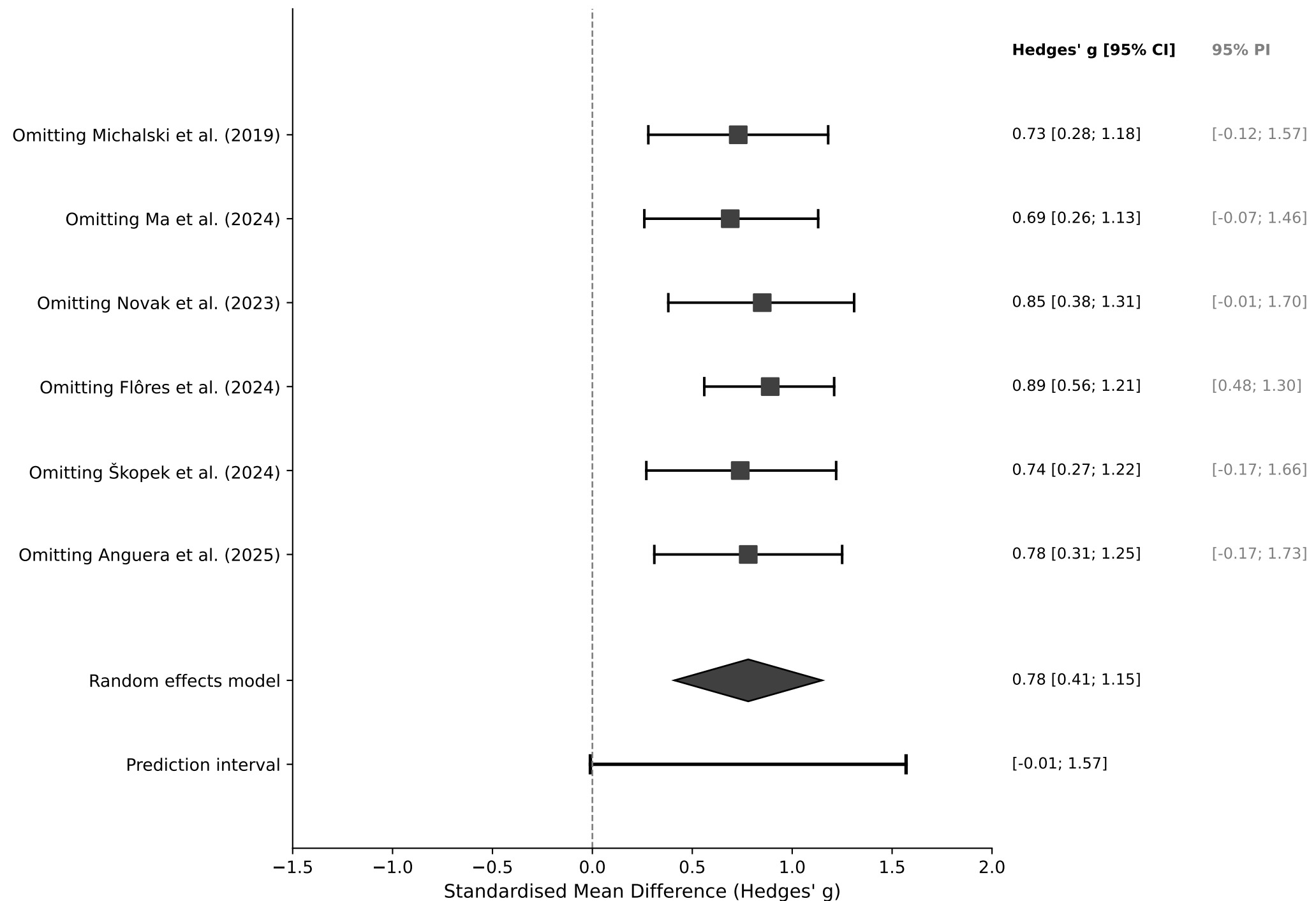

Supplement: S3 Fig — Forest plot showing the pooled effect estimate (Hedges’ g) and 95% confidence and prediction intervals when each study is omitted in turn. The overall direction of effect remained positive and statistically significant across all iterations, indicating robustness of the pooled estimate. (PDF) [file pone.0345541.s003.pdf]

## Risk-of-Bias Summary

### Overall Judgement per Study

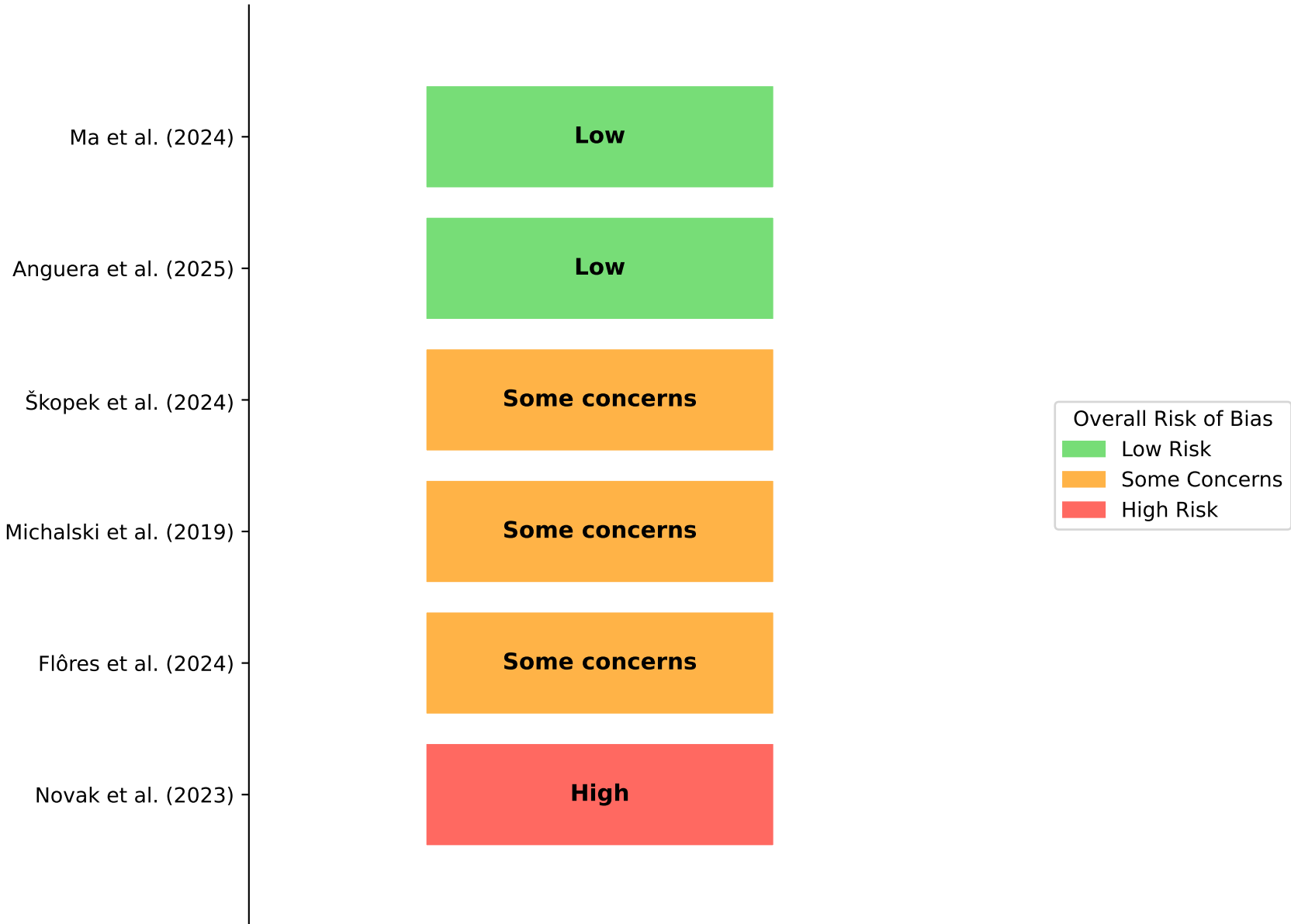

Supplement: S4 Fig — Bar chart displaying the overall risk of bias judgement for each of the six included studies: Low risk (green), Some concerns (orange), High risk (red). Assessed using RoB 2 for randomised trials and ROBINS-I for non-randomised controlled trials. (PDF) [file pone.0345541.s004.pdf]
