# Supplementary material for: Effects of virtual reality training on racket sports performance: A systematic review and meta-analysis of controlled trials
Source: PLoS One. 2026 Apr 6;21(4):e0345541. doi: 10.1371/journal.pone.0345541 (PMC13052862; doi:10.1371/journal.pone.0345541)
Supplement: S1 Table — Full electronic search strings for all five databases (PubMed, Web of Science, Scopus, SPORTDiscus, PsycINFO) with last-searched dates (15 August 2025) and number of records retrieved per database. (PDF) [file pone.0345541.s005.pdf]

S3 Table. Full Electronic Search Strategies

Database: PubMed (searched 15 August 2025)

Search Strategy:

#1 "tennis"[MeSH Terms] OR "tennis"[Title/Abstract] OR "table tennis"[Title/Abstract] OR "racket sport*"[Title/Abstract] OR "racquet sport*"[Title/Abstract] OR "ping pong"[Title/Abstract]

#2 "virtual reality"[MeSH Terms] OR "virtual reality"[Title/Abstract] OR "VR"[Title/Abstract] OR "head-mounted display*"[Title/Abstract] OR "HMD"[Title/Abstract] OR "exergam*"[Title/Abstract] OR "active video game*"[Title/Abstract] OR "virtual environment*"[Title/Abstract] OR "immersive technolog*"[Title/Abstract]

#3 "randomized controlled trial"[Publication Type] OR "controlled clinical trial"[Publication Type] OR "random*"[Title/Abstract] OR "trial"[Title/Abstract] OR "controlled"[Title/Abstract] OR "comparative study"[Title/Abstract] OR "intervention"[Title/Abstract]

#4 #1 AND #2 AND #3

Filters applied: None

Results: 89 records

Database: Web of Science Core Collection (searched 15 August 2025)

Search Strategy:

#1 TS=(tennis OR "table tennis" OR "racket sport*" OR "racquet sport*" OR "ping pong")

#2 TS=("virtual reality" OR VR OR "head-mounted display*" OR HMD OR exergam* OR "active video game*" OR "virtual environment*" OR "immersive technolog*")

#3 TS=(random* OR trial OR controlled OR "comparative study" OR intervention)

#4 #1 AND #2 AND #3

Filters applied: None

Results: 142 records

Database: Scopus (searched 15 August 2025)

Search Strategy:

TITLE-ABS-KEY(tennis OR "table tennis" OR "racket sport*" OR "racquet sport*") AND TITLE-ABS-KEY("virtual reality" OR VR OR "head-mounted display*" OR exergam* OR "active video game*") AND TITLE-ABS-KEY(random* OR trial OR controlled OR intervention)

Filters applied: None

Results: 118 records

Database: SPORTDiscus via EBSCOhost (searched 15 August 2025)

Search Strategy:

S1 TI(tennis OR "table tennis" OR "racket sport*" OR "racquet sport*")

```
OR AB(tennis OR "table tennis" OR "racket sport*" OR "racquet sport*")

S2 TI("virtual reality" OR VR OR "head-mounted display*" OR exergam* OR "active video game*") OR AB("virtual reality" OR VR OR "head-mounted display*" OR exergam* OR "active video game*")

S3 TI(random* OR trial OR controlled OR intervention) OR AB(random* OR trial OR controlled OR intervention)

S4 S1 AND S2 AND S3
```

Filters applied: None

Results: 67 records

Database: PsycINFO via Ovid (searched 15 August 2025)

Search Strategy:

```
1. (tennis or "table tennis" or racket sport* or racquet sport*).ti,ab.

2. (virtual reality or VR or head-mounted display* or HMD or exergam* or active video game* or virtual environment*).ti,ab.

3. exp Virtual Reality/

4. 2 or 3

5. (random* or trial or controlled or intervention).ti,ab.

6. 1 and 4 and 5
```

Filters applied: None

Results: 40 records

Summary of Database Searches

Database	Records Retrieved
PubMed	89
Web of Science	142
Scopus	118
SPORTDiscus	67
PsycINFO	40
**Total**	**456**
**After deduplication**	**456**

Note: Records were imported and deduplicated using EndNote X9 reference management software.
