# Supplementary material for: Effects of virtual reality training on racket sports performance: A systematic review and meta-analysis of controlled trials
Source: PLoS One. 2026 Apr 6;21(4):e0345541. doi: 10.1371/journal.pone.0345541 (PMC13052862; doi:10.1371/journal.pone.0345541)
Supplement: S1 File — Completed PRISMA 2020 reporting checklist indicating the location of each required reporting item within the manuscript. Items 15 and 22 (GRADE certainty assessment) are marked as not performed; rationale is provided in Section 4.4. (PDF) [file pone.0345541.s006.pdf]

PRISMA 2020 Checklist

Manuscript: Effects of Virtual Reality Training on Racket Sports Performance: A Systematic Review and Meta-analysis of Controlled Trials

Registration: PROSPERO CRD420251132325 | PRISMA 2020 compliant | Random-effects meta-analysis with Paule–Mandel τ^2 and HKSJ-adjusted CIs

Legend: Yes = fully addressed; Partial = needs strengthening; No = not addressed; NA = not applicable.

Item	Checklist Topic	Reported	Location in Manuscript
1	Title	Yes	Title (p. 1)
2	Abstract	Yes	Abstract contains total N=426, primary effect g=0.78 [0.41, 1.15], I^2=52%, and RoB summary.
3	Rationale	Yes	1. Introduction (p. 2)
4	Objectives	Yes	1. Introduction (end) (p. 2)
5	Eligibility criteria	Yes	2.2 Eligibility Criteria (p. 3)
6	Information sources	Yes	2.3 Search Strategy (p. 3)
7	Search strategy	Yes	2.3 Search Strategy; full line-by-line strategies provided in S3 Table (PubMed/Scopus/WoS/SPORTDiscus/PsycINFO) with last-searched dates.
8	Selection process	Yes	2.4 Study Selection and Data Extraction (p. 4)
9	Data collection process	Yes	2.4 Study Selection and Data Extraction (p. 4)
10a	Data items: outcomes	Yes	2.4 Data Extraction (outcomes) (p. 4)
10b	Data items: other variables	Yes	2.4 Data Extraction (study characteristics) (p. 4)
11	Study risk of bias assessment	Yes	2.5 Risk of Bias Assessment (p. 4)
12	Effect measures	Yes	2.6 Statistical Analysis (Hedges' g) (p. 4)
13a	Process for deciding study eligibility	Yes	2.6 Statistical Analysis (p. 4)
13b	Methods to explore heterogeneity (I^2, subgroups)	Yes	2.6 Statistical Analysis (p. 4)
13c	Sensitivity analyses	Yes	2.6 Statistical Analysis (p. 4)
13d	Synthesis approach (random-effects; Paule–Mandel τ^2; HKSJ)	Yes	2.6 Statistical Analysis (p. 4)
13e	Missing data / transformations	Yes	2.6 Statistical Analysis & Supplementary Methods (p. 4)
13f	Software	Yes	2.6 Statistical Analysis (R, meta) (p. 4)
14	Reporting bias assessment	Partial	2.6 Statistical Analysis (Funnel plot) (p. 4); k=6 insufficient for Egger's test.
15	Certainty assessment	No	Not performed. Rationale provided in Section 4.4: k=6 with substantial heterogeneity would preclude meaningful GRADE ratings beyond 'very low certainty'.
16a	Study selection (numbers)	Yes	3.1 Study Selection & Figure 1 — identified=456; screened=456; excluded=424; full-text assessed=32; reports excluded=26; included=6.
16b	Excluded studies (with reasons)	Yes	Figure 1 box lists reasons: Not randomized/controlled (n=7); Non-VR or mixed intervention (n=6); Out of scope (n=4); Wrong outcomes (n=4); Duplicate/overlapping (n=3); Full text unavailable (n=2).
17	Study characteristics	Yes	3.2 Study Characteristics & Table 1 (p. 4)
18	Risk of bias in studies	Yes	3.3 Risk of Bias & S5 Fig (p. 4)
19	Results of individual studies	Yes	3.4–3.6 & Figures 2–3 (p. 5)
20a	Results of syntheses (summary)	Yes	3.4 Overall Meta-analysis (p. 5)
20b	Heterogeneity & exploration (I^2, subgroup, sensitivity)	Yes	3.5–3.6 (p. 5)
20c	Synthesis limitations & robustness	Yes	3.6 Sensitivity Analysis (p. 5)
20d	Additional analyses (subgroups)	Yes	3.5 Subgroup Analysis (p. 5)

Item	Checklist Topic	Report ed	Location in Manuscript
21	Reporting biases (across studies)	**Partial**	3.6 & Figure 4 (p. 5); descriptive only due to k=6.
22	Certainty of evidence	**No**	Not performed. Rationale provided in Section 4.4.
23a	General interpretation of results	**Yes**	4. Discussion 4.1–4.3.
23b	Limitations of evidence	**Yes**	4. Discussion Section 4.4 (p. 4)
23c	Limitations of review processes	**Yes**	4. Discussion Section 4.4 (limitations) (p. 4)
23d	Implications	**Yes**	4.3 Practical Implications & 5. Conclusions (p. 6)
24a	Registration information	**Yes**	2.1 Protocol and Registration PROSPERO CRD420251132325.
24b	Protocol access	**Yes**	2.1 Protocol and Registration; public PROSPERO URL provided.
24c	Amendments to registration	**Yes**	No amendments reported.
25	Support	**Yes**	Funding statement in submission system.
26	Competing interests	**Yes**	Competing interests statement in submission system.
27	Data availability	**Yes**	Data Availability — OSF repository link provided (osf.io/np79q; osf.io/hdxbt).
